# Single molecule dynamics in a virtual cell combining a 3-dimensional matrix model with random walks

**DOI:** 10.1038/s41598-024-70925-2

**Published:** 2024-08-28

**Authors:** Gregory I. Mashanov, Justin E. Molloy

**Affiliations:** 1 The Francis Crick Institute, London, NW1 1AT UK; 2 Warwick Medical School, University of Warwick, Coventry, CV4 7AL UK

**Keywords:** Biophysics, Cell biology, Computational biology and bioinformatics, Physiology

## Abstract

Recent advances in light microscopy have enabled single molecules to be imaged and tracked within living cells and this approach is impacting our understanding of cell biology. Computer modeling and simulation are important adjuncts to the experimental cycle since they aid interpretation of experimental results and help refine, test and generate hypotheses. Object-oriented computer modeling is particularly well-suited for simulating random, thermal, movements of individual molecules as they interact with other molecules and subcellular structures, but current models are often limited to idealized systems consisting of unit volumes or planar surfaces. Here, a simulation tool is described that combines a 3-dimensional, voxelated, representation of the cell consisting of subcellular structures (e.g. nucleus, endoplasmic reticulum, cytoskeleton, vesicles, and filopodia) combined with numerical floating-point precision simulation of thousands of individual molecules moving and interacting within the 3-dimensional space. Simulations produce realistic time-series video sequences comprising single fluorophore intensities and realistic background noise which can be directly compared to experimental fluorescence video microscopy data sets.

## Introduction

Modern optical microscopy allows individual biomolecules to be visualized “in vitro” or in dead, chemically-fixed, cells^1–4^ but here we focus on single molecule imaging studies made “in vivo”, either in isolated live cells^5–9^ or in semi-intact tissue samples^10,11^. Such experiments allow the movements and interactions of many thousands of individual molecules to be followed in real-time within a live biological cell. Wide-field episcopic and confocal illumination methods^8^ or Total Internal Reflection Fluorescence Microscopy (TIRFM)^12^ are amongst the most commonly used approaches for high-resolution^13^ dynamic imaging in live cells. All techniques are limited by the relatively low signal-to-noise ratio of single fluorophore emission compared to background noise (e.g. cell auto-fluorescence and other noise sources). Most workers in the field use a set of criteria to confirm visualization and detection of individual fluorescent molecules: (1) Steady fluorescence level under steady illumination, (2) Single Gaussian distribution of intensities of individual spots, centered at the known intensity for single fluorophores measured under control conditions, (3) Single-step photobleaching to background intensity level with half-life that depends linearly on excitation intensity^14^. Also, the stochastic nature of single molecule movements and interactions means many thousands of individual molecules must be sampled and their trajectories, in space and time, analyzed in order to draw statistically-valid conclusions. Automatic single particle tracking by computer^15,16^ increases the spatial precision of single fluorophore localization, helps eliminate observer bias and substantially reduces the time required for analysis.

**Fig. 1:**
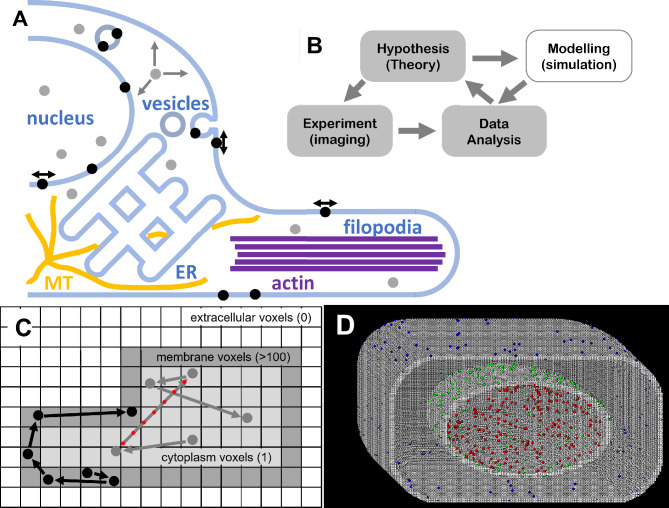
Model description. (**A**) Cartoon of cell structures with single molecules (black and grey dots) moving within the cytosol or luminal spaces (ER, nucleus, filopodia) on membranes or along cytoskeletal tracks. (**B**) Flow-chart showing the typical iterative cycle of single molecule research which usually includes simulations as an important adjunct. (**C**) Here, we show a 2-dimensional slice of the structural model at coarse resolution. The value of each byte of computer memory represents one voxel in 3-dimensional (3-D) space, that is coded to represent material and chemical properties, e.g. different cytosolic classes occupy values from 1 to 20 (light grey), membranes are in the range 200–255 (dark grey) and extra cellular regions are set to zero (white). During the simulation, molecules diffuse through the virtual space and the 3-D map is used as a look-up table of physico-chemical properties at each time-step of the simulation. Molecules may either move freely through all voxels or, more realistically, may be entrapped within one class of voxel (here, black molecules move within membrane, grey within cytosol). (**D**) The model expanded to 3-D: A yeast cell (0.8 × 0.6 × 0.6 µm^3^) with simplified elliptical nucleus (diameter 400 nm, flattening = 0.8) populated by cell membrane molecules (blue circles), nuclear membrane molecules (green circles), and nucleoplasm molecules (red circles). Cell and nucleus membranes are 3 voxels thick.

Studies of fluorescently-tagged molecules present at the plasma membrane, within the cytoplasm^17^, nucleus^18^ or other cellular compartments^9,11,19^ (Fig. 1A) have increased rapidly over the last two decades. Purpose-built computer models have been applied to validate both methods of data analysis and conclusions^16–18,20–22^ drawn from the results. More generalized models have also been created to simulate movements and chemical interactions between individual molecules within a “virtual cell framework”^23–25^. Some computer models produce results in the form of simulated sequences of images (i.e. movies)^16,26,27^ that closely-match experimental video data sets and this presents an important part of the research cycle (Fig. 1B)ref ^28^ as the analytical tools can be validated against known truths. To date, most models have been limited to simple, fixed, shapes with geometrical constraints built within an isotropic volume or planar surface^18,24,26,29^. However, biological cells have complex, dynamic, architectures, consisting of irregularly shaped organelles (Fig. 1A) and subcellular components built of different materials (membranes, cytoskeletal proteins, large protein complexes) each with distinct physical–chemical properties. Here, we have created a realistic cellular space in which a 3-dimensional framework of arbitrary structures is mapped in computer memory with each 8-bit voxel (memory location) coded to represent the physical–chemical properties (Fig. 1C). Diffusion of individual molecules within this virtual space is simulated (with floating-point precision) using a Monte Carlo process and interactions between molecules and with the virtual architecture is checked at every computational time-step using the voxel values within the memory-mapped architecture as a lookup table. The fidelity of the model is limited simply by computer memory although coarse-graining of the structural model can be applied if very large cells or tissues need to be simulated. In the current model, if we wish to simulate an isolated, cultured mammalian cell of dimension 10 μm × 20 μm × 5 μm then ~ 10 Gb of computer memory would allow a voxel size of 100 nm^3^ (i.e. linear dimension 4.5 nm) which is approximately the volume of a single actin protein monomer (molecular weight, MW, 42 kDa). If we wish to model an, *S. pombe*, yeast cell using the same amount of computer memory the voxel dimensions could be proportionately reduced to 10 nm^3^ (sufficient to approximate the domain shape of individual proteins). However, our freely-diffusing, fluorescently-tagged molecules must correctly sample the voxel topology as they move through the virtual cell. A typical protein (e.g. MW 42 kDa) with cytoplasmic diffusion coefficient, D = 3 × 10^6^ nm^2^⋅s^−1^ would take t ~ 1 μs to diffuse across a voxel of linear dimension R = 4.5 nm (where t = R^2^/6D). So, in order to sample the voxelated space 30,000 computational cycles (of time-step Δt = 1 μs) would be required to generate each 30 ms video frame. However, if we apply linear interpolation methods and check the properties of perhaps up to 5 voxels that a molecule might pass through during a single time-step then we can produce satisfactory sampling of the voxel topology with time-steps of ~ 30 us (i.e. 1000 iterations per video frame).

In this paper, we describe how cellular architectures can be created in computer memory, and how a Monte Carlo continuum model can be applied to the voxelated space to give realistic simulations of individual molecules interacting with each other and with cellular features that have distinct physical/chemical properties. We then describe how simulations are converted to data sets that closely mimic experimental videos generated by different optical imaging methods and camera systems. Finally, we compare our model output with real experimental data sets and evaluate movement of molecules in cytoplasm, cell membranes and within tubular networks and also show how intracellular dynamics (filopodial trafficking and endocytosis) can be simulated by updating the virtual cell architecture held in memory at appropriate time intervals during the simulation. Algorithms and code examples are given in the Supplementary Material to illustrate how the model works.

## Methods

### Overview

The model consists of a “simulation engine” and “model output” routines that together produce realistic fluorescence video microscopy data that is derived from known input starting values. As the model runs, complex emergent behaviors can be generated. The model system can be made as simple or complex as the experimenter requires in order to test new ideas, generate new hypotheses and validate downstream analytical approaches (i.e. image analysis methods can be tested against a ground truth). The model was written in Borland C++, but the general approach could be ported to *MatLab*, *Python*, or other computer language.

#### Simulation engine

The approaches used for numerical modeling of diffusive and motorized movements of molecules and their interactions with each other and with structures in a simple virtual cell have been described previously^26^. In brief, at every time step (Δt) in the Monte Carlo simulation the following computations and logical tests are performed:

- Freely diffusing molecules move a random distance in Cartesian space (x,y,z) using a Gaussian-distributed random number generator to simulate a Brownian walk. Motor-driven molecules (e.g. myosin, kinesin or dynein) move a fixed distance per time-step (Δt) along a vector to simulate motion along a cytoskeletal track (e.g. actin filament or microtubule). Many different molecules can, in principle, occupy the same voxel since their individual coordinates (x,y,z) are always held with floating-point precision.
- Coordinates are checked to test if the molecule approaches within a characteristic collision distance of any other molecule and a subsequent test is made for stochastic binding probability or dissociation if the molecules are already in a bound state.
- Tests are then made to check for molecular interactions within the voxel lattice elements along a linear, interpolated, path made during the time step (red circles on the arrow on Fig. 1C). The byte value of the voxel is used to lookup the voxel properties (e.g. viscosity, medium type, and properties modulating objects movements and interactions) and then suitable tests are made for stochastic binding/unbinding or reflections at voxel element boundaries. This permits interactions with cellular structures (e.g. cytoskeletal elements) and can also constrain motion of diffusing (Supplementary Material Code Example 5&6) or motorized molecules within a particular cellular compartment (e.g. cytosol, membrane or cytoskeletal filament track).
- Photobleaching probability of the fluorescent tag is computed based on an exponentially-distributed random function.
- The lattice structure, held in computer memory, is updated and remodeled in order to simulate cellular dynamics such as membrane protrusion, vesicle movement, cytoskeletal polymerization etc.

All of the Monte Carlo modeling computations are made and results are always stored using floating-point numbers. A rounding algorithm is then used to index the discretely sampled lattice voxels held in computer memory (memory addressing is made using a “pointer” in C + +). The byte value at the voxel address is then used to index a set of physical–chemical properties held in a 2-dimensional look-up table (i.e. a “pointer to a pointer”).

### Creating the 3-dimensional virtual cell

Computer memory is allocated as a 3-dimensional unsigned byte array to represent “voxels” which are volume elements, analogous to “pixels” in a 2-dimensional image (Fig. 1C). The array dimensions (x,y,z) define a rectanguloid volume which encompasses the virtual cell surrounded by isotropic extracellular medium. The voxel size (scaling) is made appropriate for the purpose of the simulation; for example, a 2 nm unit length (8 nm^3^ voxel volume) might be used for simulating tightly curved membrane structures within a yeast cell while 10 nm unit length (1000 nm^3^ volume) might be sufficient for studying dynamics of molecules within the cytoplasm of a larger mammalian cell. Memory requirement in gigabytes is given by cell volume (measured in μm^3^) divided by the cube of voxel unit dimension (nm). So to represent a mammalian cell with dimensions (x,y,z) of 10 × 20 × 5 µm^3^ (= 1000 μm^3^) using voxels of 10 nm unit length we require 1Gbyte; whereas a higher spatial resolution of 2 nm unit length would require 125 Gigabytes of memory.

Each byte of computer memory holds a code number (0–255) that is used to index a table of physical–chemical properties. For convenience values are grouped into broadly similar classes so “membrane lipids” are in the range 100–255 and “solutes” (e.g., cytoplasm, nucleoplasm, vesicular lumens, or extracellular medium) in the range 0–10 (Fig. 1C).

Memory is allocated and the 3D array initialized to zero (extracellular medium) then cell compartments, cytosol, lipid membrane and cytoskeletal elements etc. are created by adding a combination of geometrical and more complex predefined shapes and using standard image processing binary morphology operations to erode or dilate in order to hollow-out or thicken the structures.

### Plasma membrane

Simple cell shapes can be defined using a symmetrical prolate ellipsoid to represent a yeast or bacterial cell^30^ or a low-dimensional outline can be used to represent the more complex morphology of a mammalian cell^11^. The enclosed volume is initialized to represent plasma membrane (here a value of 200). A binary “erode” function is used to hollow-out the cell and fill the interior with cytoplasm (value 1) leaving a single voxel layer to represent the outer plasma membrane a subsequent binary “dilate” operation (also called a “close” operation) seals gaps in diagonal connectivity. Further dilate operations can be used to thicken the membrane if for instance the voxel unit size is < 4 nm (Fig. 1 C&D and (Supplementary Material Code Example 1&2).

### Sub-cellular organelles

Hollow spheroids, oblate and prolate ellipsoids and tubular morphologies can be added within the cytoplasm volume using Cartesian geometry and floating-point arithmetic. Bounding voxel coordinates are defined by rounding to the nearest integer value (Supplementary Material Code Example 3). To create realistic tubular networks with characteristic persistence length, Lp, ellipsoids are extruded along a variable solid angle, Δ*θ*, at each extrusion step, Δd, using a Gaussian distributed random number generator, *GRand()* (with unit standard deviation) to modulate direction Eq. (1), where:

1 $$\Delta \theta = \cos^{ - 1} \left( {e^{{\frac{ - \Delta d}{{L_{p} }}}} } \right) \times GRand()$$

Giving the average change in extrusion vector angle per extrusion step Eq. (2)

2 $$\left\langle {\cos \Delta \theta } \right\rangle = e^{{{\raise0.7ex\hbox{${ - \Delta d}$} \!\mathord{\left/ {\vphantom {{ - \Delta d} {L_{p} }}}\right.\kern-0pt} \!\lower0.7ex\hbox{${L_{p} }$}}}}$$

In most cases, tube extrusion is terminated on contact with any non-cytoplasm voxel (see Movie S1). However, the same algorithm can be used to build filopodia projecting from the cell body into extracellular space in which case extracellular voxels are replaced with plasma membrane and cytoplasm voxels over a given extrusion distance (Fig. 1C).

Voxels bounding the internal tubular and ellipsoidal structures are set to appropriate “membrane lipid” voxel values (in the range 100–255, see above) and inner volume voxels are filled with uniform “cytosol” class values (range 1–10). For further details and refinements see Code Example 4 in Supplementary Material.

Seeding with fluorescently–tagged molecules: An object-orientated computing approach^26,31^ is used to represent each individual protein molecule of interest as an object of a certain “class” with associated properties (e.g. color of fluorescent tag, diffusion coefficient) and “methods” (or functions) that describe specific chemical or physical properties and more complex behavior e.g. motor molecules that bind and move on the cytoskeleton^19,22^. A random number generator was used to seed molecules at random starting coordinates within valid regions (voxels) of the cell or sub-cellular compartment (e.g. membranes, cytosol, lumen). To speed initialization, small sub-cellular compartments are filled while they are created to ensure that molecules occupy valid starting locations (see Fig. 1D). In some cases, untagged protein molecules (e.g. tag fluorescence = 0) are placed at fixed starting locations (e.g. diffusion coefficient = 0) to simulate static filamentous structures, such as actin filaments or microtubules. The principles used to build the cytoskeleton are analogous to building tubular membrane networks, described above.

### Dynamic cellular structures

Dynamic structures (e.g. filopodia) are simulated by either adding or removing voxel elements at the tip by updating the “membrane” voxels and filling the newly enclosed or newly exposed voxel regions with cytoplasm or extracellular medium). Vesicle diffusion is simulated using a Gaussian random number generator^32^ to produce a Brownian walk with diffusion coefficient, D, given by Eq. (3) ref^26^:

3 $$D = \frac{{k_{B} T}}{6\pi \eta r}$$

where, k_B_T is thermal energy, r, vesicle radius, and η, cytosolic viscosity. To reduce computation time, the (x,y,z) centroid location is held as a floating-point value and memory updates are made only when the centroid moves by > 1 voxel unit distance (see Supplementary Material, Code Example 3). To model the thermal motion of ellipsoidal organelles (e.g., mitochondria or Weibel–Palade Bodies^9^), rotational diffusion is computed and variation in the long-axis angle of the vesicle is updated and stored and memory voxels updated in the same way as for translational motion (above).

Membrane fusion events are modeled in a probabilistic manner whenever vesicular membrane collided with plasma membrane. If the two membranes fuse, membrane proteins then diffuse between membrane compartments and molecules within the vesicle either enter or exit the extracellular space.

### Single molecule movements and interactions

Thermal motion of individual molecules^26^ is computed using a Gaussian-distributed random number generator (with unit standard deviation) to give a diffusive random walk displacement along each axis (x, y, z) in Cartesian space at each simulation time step, Δt Eq. (4):

4 $$\Delta x,\Delta y,\Delta z = \sqrt {2D\Delta t} \times GRand()$$

At each time step, the linear path taken through voxel space is checked to ensure every intervening voxel had the correct value, for example, membrane-proteins must move only through membrane voxels and path direction should follow the nearest membrane voxel path (Supplementary Material Code Examples 5&6). To ensure molecules remain correctly located within or moving on sub-cellular organelles all molecules associated with that organelle (vesicle) are moved the same discrete distance (voxel step) whenever the organelle location is updated (as described above). We use an algorithm, called “move to nearest valid voxel” (Code Example 9 in Supplementary Material), to ensure the correct localization of molecules during membrane remodeling (e.g. filopodial growth/shrinkage). In this case the location of each molecule is checked in voxel space and, if needed, the molecule is moved to the nearest voxel of the correct medium. See Movie S2 simulating diffusion of membrane molecules during vesicle fission. For simulations of single molecule diffusion within either cytosol or membrane the diffusion coefficient, D, for each molecular species is entered explicitly using experimentally or theoretically determined values.

Whenever molecules collide with each other, binding and dissociation rules are applied knowing the respective physical/chemical properties^26^. Binding probability depends on collision distance and also a binding rate constant which are evaluated to a probability per time interval, Δt. Molecular complexes (e.g. homo-dimers, hetero-dimers or protein–ligand pairs) move as a single object, governed by the slowest diffusion coefficient and if one member is lipid bound (membrane protein) the complex then remains in membrane. Probability of complex dissociation depends on the dissociation rate constant expressed as a probability per Δt time period and tested using a uniformly-distributed random number generator (Rand())^26^.

Multi-molecular interactions like the assembly and disassembly of protein complexes at the plasma membrane can be simulated as molecules are first allowed to aggregate and form clusters and then rapidly dissociate to monomers when the on-rate constant (binding) is set to zero. This type of simulation may be found useful to mimic spontaneous formation of signaling complexes or experimental manipulations like opto-genetic or caged-compound experiments (see Movie S3). Cytoskeletal dynamics of assembly “growth” and disassembly “catastrophe” of actin filaments and microtubules can be simulated as diffusing monomers stochastically bind/unbind at the growing/shrinking filament ends (see Movie S4).

#### Model output

Output data is documented either in raw binary or as a comma-delimited, ASCII file (“.csv” format) listing x*,y,z,* coordinates, bound state, fluorescence, and so on at every time point or as mock fluorescence video microscopy movies that closely mimic single fluorophore imaging experiments (Fig. 1B). The algorithms and equations used to generate sequences of synthetic fluorescence video microscopy movies under different illumination conditions have been described previously^26^. Briefly, for each video image illumination intensity is calculated for each fluorescent molecule according to its *x,y,z* coordinates and given illumination profile (e.g., TIRFM illumination with Gaussian beam profile). The mean number of emitted “photons” for each fluorophore is proportional to illumination intensity and photon noise is proportional to square-root of the mean value. In the simulation, each fluorophore emits photons until it stochastically photobleaches (as described above). Additionally, fluorophore “blinking” can also be simulated as stochastic fluctuations between ON/OFF states (See screenshots of model settings in Supplementary Materials). Photons emitted by individual fluorophores are centered at the appropriate *x,y* location on the mock image and spread over a Gaussian-shaped point-spread function (PSF) which matches the experimental imaging system (See text and Fig. S1 in Mashanov, 2014^26^). Additional noise contributions from the camera and sample auto-fluorescence are also summed onto each mock image. The accumulated output of several model time-steps (2-to-1000) is summed onto the virtual x,y image to simulate frame integration times that are typical of video microscopy camera systems (e.g. 20 or 40 ms per frame) yielding “mock imaging rates” of 50 or 25 frames per second.

Because the fluorophore locations at all time points are stored with floating point precision, output from the simulations are well-suited to DSTORM^33^ and other forms of super-resolution single fluorophore localization analysis. More advanced microscopy approaches like the use of polarized illumination and fluorophore dipole orientation^4,34^, STED, MinFlux^35^, fluorescence correlation spectroscopy (FCS)^36^ and other methods could be added to the model as different “model output” using the same underlying simulation engine. For example, FCS data can be simulated by narrowing the illumination PSF to ~ 0.2 μm (or ~ 0.05 μm for STED) and increasing the imaging rate to 10,000–100,000 fps and data can be output in ASCII format as intensity vs time, suitable for downstream auto- or cross-correlation analysis.

## Results

### Curved and planar membranes

**Fig. 2:**
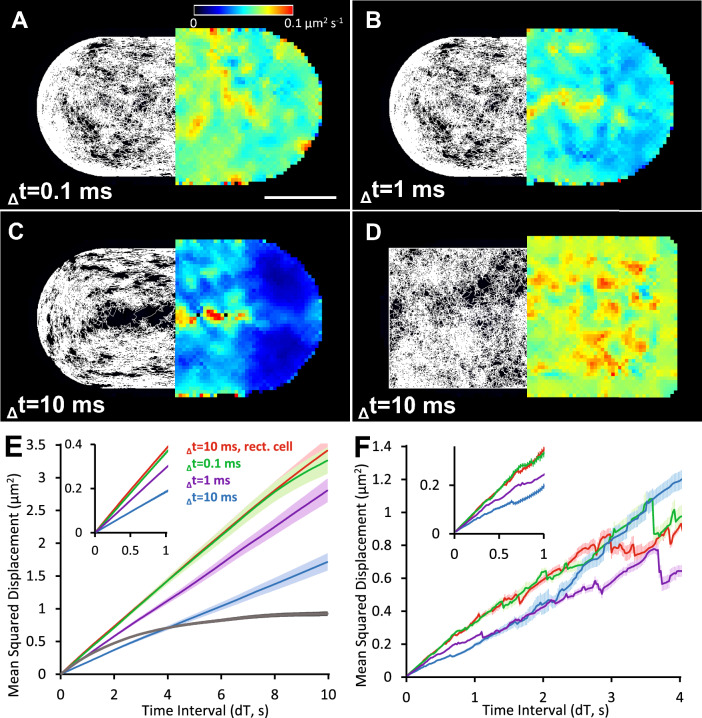
Single molecules diffusing within plasma membrane. (**A–C**) show the pattern of *xyz* trajectories projected onto the *x,y*-plane (left) and mobility heat maps (right) for rounded cell shapes and (**D**) shows the results in a rectangular cell of the same dimensions. Simulation time steps were _Δ_t = 0.1 ms, 1 ms, and 10 ms as shown and the diffusion coefficient, D, was 0.1 µm^2^⋅s^−1^ in all simulations. (**E**) MSD vs dT plot (± SEM) for trajectories shown in panels (**A–D**), where dT is the time interval between measurements^36^. Averaged MSD was calculated using real *x,y,z* coordinates of each single molecule grey line—small, rounded cell (radius = 0.5 µm, simulation step time, Δt = 0.1 ms, not shown). Inset – the slope of the initial part of MSD vs dT plot (up to 1 dT = 1 s) was used to calculate mobility values (See Table 1). (**F**) as (**E**), but instead of using known *x,y,z* coordinates, the mock movies were tracked using custom video tracking software (see main text) and the *x,y*-trajectories were used to build the MSD vs dT plots (See Table 1**).**

Single molecule diffusion was evaluated in a simulated yeast cell^30^ (radius, R = 2 µm) with a total model volume 8 × 4 × 4 µm^3^, voxel unit size 5 nm, membrane thickness 3 voxels. The cell was populated with 100 membrane-proteins (surface density ~ 1 µm^−2^) with diffusion coefficient D = 0.1 µm^2^⋅s^−1^ close to experimentally measured values^5,7,37^. The effect of varying the model time-step (Δt) on measured diffusion coefficient was tested at Δt = 0.1 ms, 1 ms and 10 ms, using a fixed mock imaging rate of 50 frames per second (exposure interval 20 ms). Diffusion coefficients were then computed either directly from the known x,y,z trajectories generated by the model (i.e. the ASCII data file output) or by analyzing the simulated, mock video sequences using our custom-written single particle tracking software^16^ and mobility heat maps were generated as described earlier^38^. The simulation was also conducted using a rectangular cell that bounded the modeled volume (see Fig. 2A–D, Table 1 and Movie S5). For the spherical cell, short Δt values gave good estimates of the true diffusion coefficient however the longer model step delay times meant that the diffusional path deviated significantly from motion in a single plane so estimates of D were subject to error due to projection of 3D motion into two dimensions. The long-range movements were further restricted in a smaller tightly curved (R = 0.5 µm) cell (Fig. 2E, grey line). The surfaces of the rectangular virtual cell meant that observed molecules all diffused in a single x–y plane and at the longer, Δt = 10 ms, still give a reasonable estimate of D. Single fluorophore tracking in vitro^16^, can have a resolution as good as one nanometer accuracy^39^, while tracking in live cells is usually slightly worse at around 20 nm resolution Fig. 2E&F.

**Table 1:** Simulation of single molecule diffusion in curved membranes using different model time-step values, Δt with diffusion coefficient, D = 0.1 µm^2^⋅s^−1^ and mock video frame rate = 50fps.

	Rounded cell (8 × 4 × 4 µm^3^, R = 2 µm)			Rectangular cell (8 × 4 × 4 µm^3^)
Δt | mean Δx, Δy, Δz	0.1 ms | 7.7 nm	1 ms | 24.5 nm	10 ms | 77.5 nm	10 ms | 77.5 nm
Estimated D (µm^2^⋅s^-1^) From known *x,y,z*-trajectories	0.091	0.074	0.046	0.094
Estimated D (µm^2^⋅s^-1^) Tracking of mock video movies	0.093	0.071	0.056	0.094

### Relatively narrow, tubular membranes

**Fig. 3:**
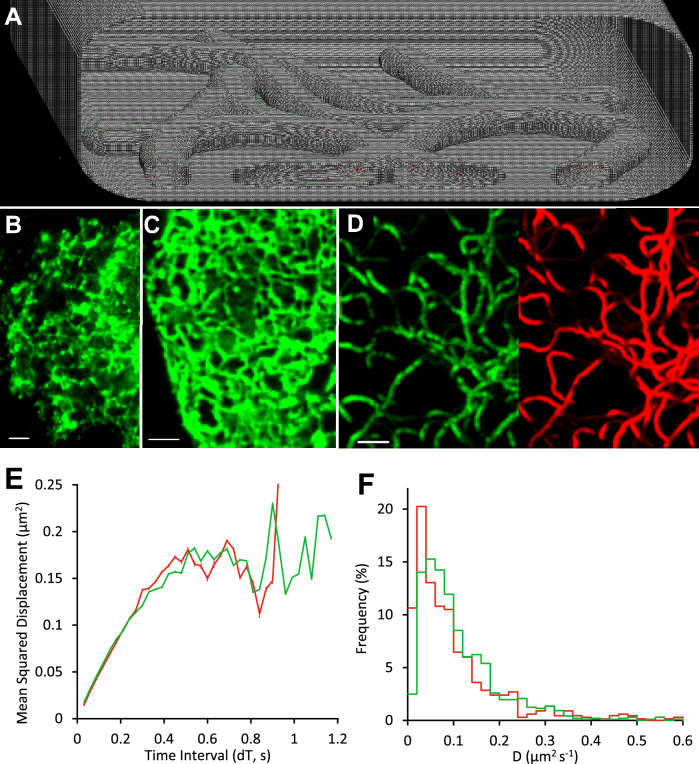
Single molecules diffusing within a tubular network. (**A**) Model: “virtual cell” with an interconnected tubular network (tube diameter = 0.2 µm). Green dots are molecules embedded in the tubular membranes; Red dots are molecules diffusing within the luminal space of the network. Experimental Data: (**B**) SD-projection image showing GFP-tagged nicotinic receptors retained in the ER of the HEK293 cell. (**C**) SD-projection image of KIR3.1-GFP potassium ion channels in the ER of a CHO cell. Scale bar 2 µm. (**D**) Model: Simulated SD-projection image showing fluorescent molecules diffusing (D = 0.2 µm^2^⋅s^−1^) in the tubular membranes (green, left side) and fluorescent molecules (D = 2 µm^2^⋅s^−1^) moving within the tube lumen (red, right side) Scale bar 2 µm (see Movie 6). (**E**) MSD vs dT plots produced by tracking the paths of 667 individual fluorophores for experimental data (nicotinic receptors, shown in panel (**B**) (red line), and 963 simulated membrane-localised molecules (green line, shown on left in panel (**D**). (**F**) Distribution of diffusion constants, measured from the initial gradient of MSD v dT plots of individual molecules; nicotinic receptors in HEK293 cell (red line); virtual cell (green line). Note: same data set as E.

An interconnected network of endoplasmic reticulum was simulated as membrane tubes of 200 nm diameter (see Methods) (Fig. 3A and Movie S1). Membrane proteins (D = 0.2 µm^2^⋅s^−1^) and intra-luminal protein molecules (D = 2 µm^2^⋅s^−1^) were seeded into the model and the simulation generated a mock video sequence at 25 fps (Movie S6). To make the data more closely resemble experimental data sets, we generated images that mimicked two-color, epifluorescence wide-field microscopy. In addition, we simulated a commonly used technique called “fluorescence recovery after photobleaching” (FRAP) by instantaneously-bleaching all the molecules in one half of the cell (Movie S6). The simulation showed characteristically rapid fluorescence recovery for molecules in solution (where D = 2 μm^2^⋅s^−1^) and slow recovery for the membrane proteins (D = 0.2 μm^2^⋅s^−1^). In addition, and as expected, molecules remained in their correct cellular compartment.

We compared the simulations to previous experimental studies in which we conducted TIRFM imaging experiments using GFP-tagged proteins that were localized to membranes or in the lumen of the endoplasmic reticulum^11,37^. Single images, called “SD-projections”^11^ were generated by computing the standard deviation of pixel intensity measured over a short section of movie data (lasting 2 s). SD-projection images of cells with endoplasmic reticulum networks containing GFP-tagged nicotinic receptors and potassium channels (KIR-6.2-GFP) are shown on Fig. 3B&C. To simulate this particular experiment the photobleaching rate was 0.1 s^−1^, the imaging mode was changed to TIRFM, and the imaging rate increased to 33 fps.

The experimental data (Fig. 3B&C) and simulated data (Fig. 3D) show a heterogeneous “patchwork” appearance (green) that is created by membrane associated molecules moving a few microns during the averaging period. Whereas, faster-moving luminal molecules (red) produce a homogeneous background “blur” that reveals the path of the ER lumen (Fig. 3D).

Plots of mean-squared particle displacement vs. time interval (called “MSD vs. dT” plots^40^) should be linear for free diffusion (i.e. a Brownian walk) but show curvature typical of anomalous diffusion (Fig. 3E). This is found both in simulations and real data sets recorded for nicotinic receptors moving on ER membranes of HEK293 cells^11^. Our model shows the easiest explanation for the data is that tube geometry limits long-range movement to a single axis (i.e. pseudo 1-dimensional) so the gradient of the MSD vs dT graph falls because the dimensionality of the diffusive motion is reduced at longer times (dT) and correspondingly longer distances. The distribution of measured and simulated apparent diffusion coefficients was also similar (Fig. 3F), but the fraction of “immobile” molecules (defined as D < 0.02 µm^2^⋅s^−1^) was higher in the real experiment (10.6% versus 2.5%). The diffusion coefficient, estimated from the MSD vs dT plot using the first 15 data points (i.e. dT ranges from 0–0.45 s) was ~ 0.1 µm^2^⋅s^−1^ for simulated data, compared to ~ 0.085 µm^2^⋅s^−1^ for nicotinic receptors-GFP. The apparent value of D derived from the initial gradient of MSD vs dT plots (where gradient should equal 4D), assumes diffusion in 2-dimensions, and does not agree with the model parameter (D = 0.2 µm^2⋅^s^−1^) because long-range movements are limited in the narrow and curved tubules which changes the dimensionality of the diffusion equation. So, our model correctly simulates solution and membrane proteins moving freely within the lumen and the membrane of intracellular tubular networks (ER) and importantly reveals that downward curvature in the MSD vs dT plots (indicative of anomalous diffusion) seen in experimental data sets can be explained simply from changes in dimensionality as motion observed at short times is 2-dimensional whereas at longer times it is effectively 1-dimensional.

### Motorized transport in filopodia

**Fig. 4:**
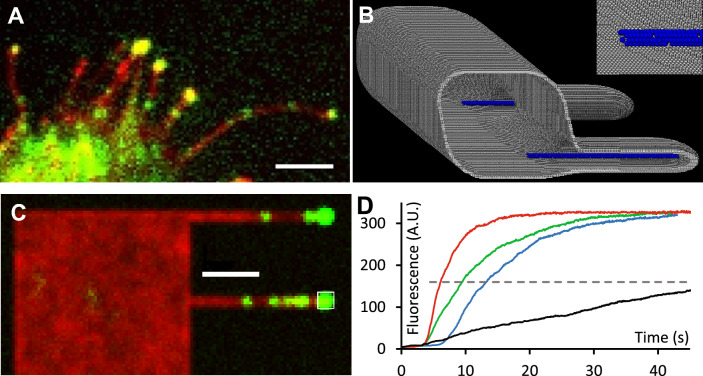
Single molecule transport in filopodia. (**A**) Experimental TIRF image of a HeLa cell transfected with myosin-10-GFP. Small green spots are individual molecules of myosin-10 moving towards the tips of filopodia which contain clusters of ~ 50 myosin-10 molecules at their tips (bright green spots). (**B**) Cross-section of a virtual cell with rounded edges (R = 0.2 µm), with simulated filopodia (Ø = 0.2 µm, L = 1 µm). Cell membrane thickness was 3 voxels and the simulated volume was 1 × 6 × 0.6 µm^3^. Sharp edges between the tubes and cell body were smoothed using the vertices method (see Supplementary Material). Inset panel shows the actin filament bundle (blue circles) inside the filopodium. (**C**) Model data showing a larger simulated volume (6 × 6 × 1 µm^3^) that generates an SD-projection image from a mock video sequence simulating the movement of individual myosin-10 molecules (small green spots) moving along actin bundles towards the filopodia tip (large green spots). The red image showing the cell shape was generated by averaging the images of red fluorescent molecules diffusing in the cell membrane over the whole image sequence. (**D**) The rate of myosin-10 accumulation at the tips of filopodia. The rate was measured as a time (T½) needed to reach the 50% of fluorescence maximum (grey dashed line) at the filopodia tips (0.5 × 0.5 µm^2^ area, white rectangle shown in (**C**). The rate depended on both the myosin-10 walking velocity (T½ ≈ 13.3 s, blue line, and T½ ≈ 9.6 s, green line, at speeds 10 and 20 steps s^−1^ respectively) and the length of actin bundle protruding out of the filopodia entrance. The rate of accumulation increased to T½ ≈ 6.1 s (20 steps s^−1^, red line) if the actin bundle was protruding 1 µm inside the cell body from filopodia shown in (**B**), and the rate decreased to T½ ≈ 50 s (black line) if the bundle was 0.5 µm shorter the length of filopodia. Scale bars are 2 µm.

We simulated motion of the actin-based molecular motor, myosin-10 that is known to traffic along actin bundles within filopodia, moving away from the cell body towards the filopodial tip^41,42^ (Fig. 4A).

It has been shown that myosin-10 moves across the cell via a combination of 3-dimensional, cytosolic diffusion, plasma membrane associated, 2-dimensional, diffusion and finally, 1-dimensional active translocation within the filopodial region^19^. Here we simulated the movement of myosin-10 molecules by giving them attributes to enable binding and dissociation from plasma membrane and actin filaments and ability to undergo motorized movement along actin and free diffusion within cytosol and membrane compartments. By choosing realistic parameter values we were able to closely mimic our experimental observations (Fig. 4A–D) and Movie S7). This type of simulation helps to validate hypotheses and also leads to testable predictions of what might be expected to happen when simulation parameters are changed. For example, we can change the length of the actin filament bundle protruding from the entry to the filopodium to specifically test if it acts like an aerial or funnel to recruit myosin molecules into the filopodium (Fig. 4D).

### Moving vesicles and exocytosis

**Fig. 5:**
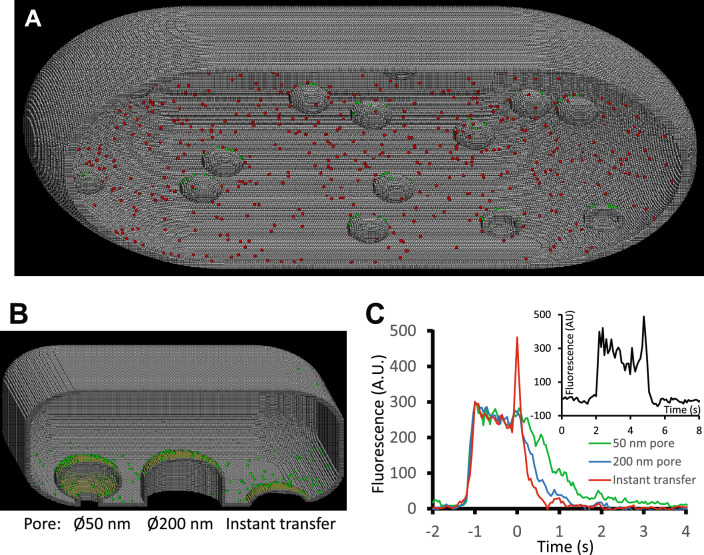
Vesicle movements and exocytosis. (**A**) Cross-section of prolate-ellipsoidal “cell” with long axis 1.8 µm and short axis 1 µm, membrane thickness 3 voxels containing vesicles of diameter = 100 nm. Red spheres represent single molecules in the cytoplasm, green spheres represent molecules in the vesicle membrane. (**B**) Cross-section of a synthetic membrane bound volume showing vesicles fused to the plasma membrane via a 50 nm diameter tube (left), a vesicle-width (200 nm diameter) tube (center) and a vesicle that fuses via direct contact with the plasma membrane (right). (**C**) Main graph shows fluorescence time-course of modelled exocytosis region averaged over ten simulated fusion events, synchronized to docking time (defined as t = −1 s). Note decay rate increases when fusion tube diameter was increased from 50 to 200 nm (green vs blue lines). Fluorescence decay was significantly faster when molecules were instantaneously transferred from the vesicle to the cell membrane (red line). Furthermore, a fluorescence spike was observed at the time of fusion because molecules in the vesicle membrane (located ~ 100 nm above the coverslip surface) moved rapidly to the basal cell membrane, where TIR illumination intensity is higher. A similar spike in fluorescence was observed in experimental data obtained using TIRFM illumination (insert). The model indicates that observation of a fluorescence spike and fast decay rate is consistent with rapid vesicle fusion. The finite delay between vesicle “docking” and fusion requires a rate-limiting step occurring before fusion pore formation.

The ability of the model to simulate single molecule dynamics in moving and transforming membrane structures was tested in the simulation of constitutive exocytosis^37,43^. This dynamic process includes several stages: vesicle docking, fusion and then spread of delivered molecules at the plasma membrane. Exocytotic vesicles have small diameter (100–200 nm) and the whole process is complete within a few seconds, so it is difficult to establish the details of the fusion process; for instance whether vesicles form a long-lived pore with the plasma membrane or instantly collapse at the moment of fusion. Here we compared published fluorescence video imaging records of constitutive exocytosis^37,43^ with simulated data sets in which vesicle and plasma membranes either came together and rapidly fused or first needed to form an intermediate, docked state, that then underwent fusion either via a narrow fusion pore or rapid membrane leaflet fusion and lipid mixing (Fig. 5 and Movie 8, 9).

### Mobility patterns on membranes with lipid rafts

**Fig. 6:**
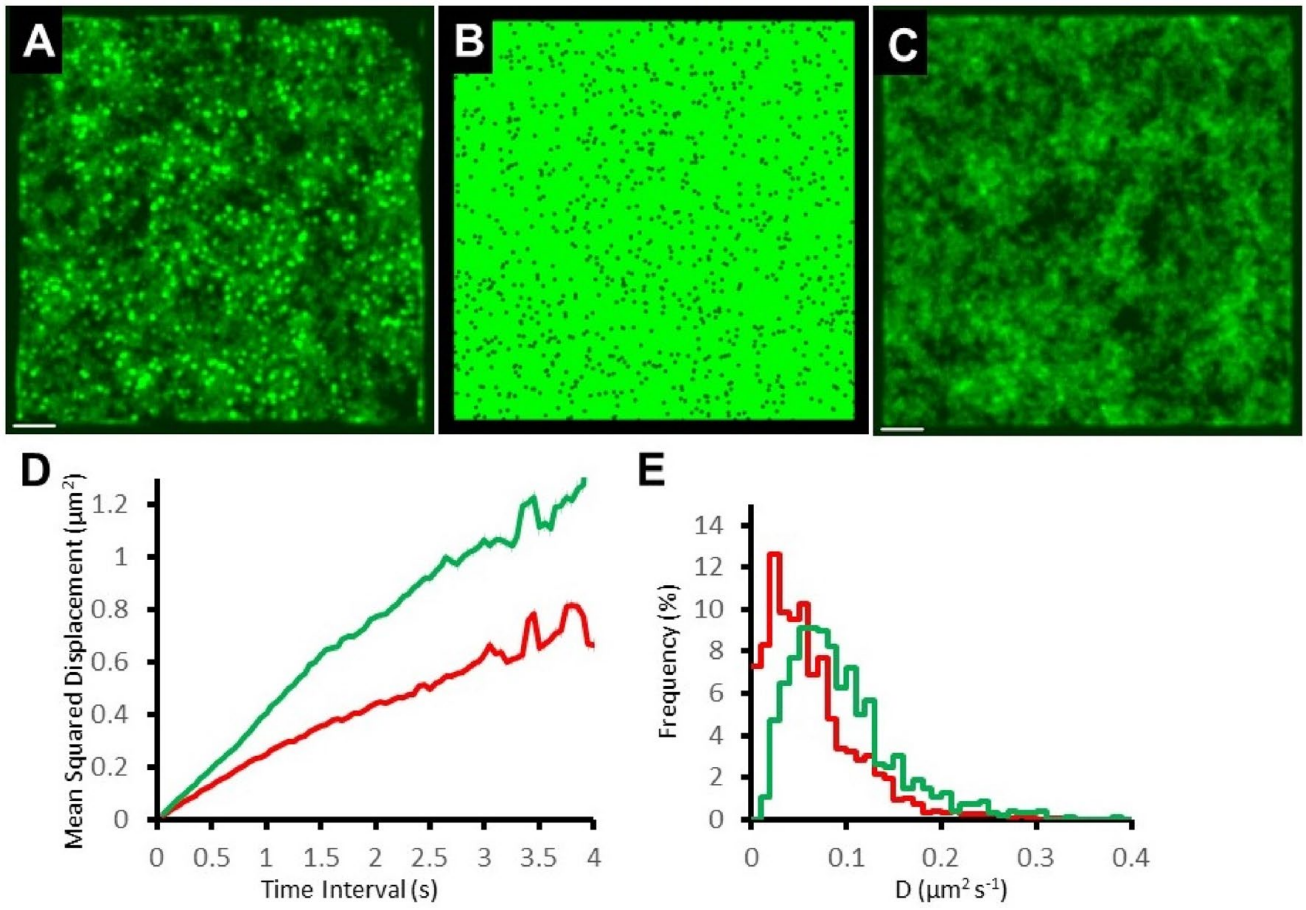
Single molecule movements in the presence of lipid rafts. (**A**) Projected SD image generated from a movie sequence (500 frames, 20 fps) simulating fluorescent molecules moving at K_diff_ = 0.1 µm^2^⋅s^−1^ in cellular membrane (20 × 20 × 2 µm^3^, voxel size 5 × 5 × 5 µm^3^) containing 1000, randomly positioned, circular lipid rafts, Ø220 nm (surface area 400 µm^2^, 10% raft coverage, viscosity × 10 of surrounding membrane). (**B**) Thresholded map showing the distribution of lipid rafts on the cell membrane shown in (**A**). (**C**) Projected SD image generated using the same conditions as in (**A**), but without lipid rafts. Scale bars are 2 µm. (**D**) MSD vs dT plots produced by tracking the paths of 1195 individual fluorophores moving on the membrane containing lipid rafts (red line) and 958 molecules moving on plain membrane (green line). The linear fit of the first 40 data points (up to 2 s time interval) gives K_diff_ = 0.056 and 0.102 µm^2^⋅s^−1^ respectively. (**E**) Distribution of diffusion constants, measured from the initial gradient of MSD v dT plots of individual molecules; cell with lipid rafts (red line); plain membrane (green line). Note: same data set as **D.**

The ability of the model to investigate experimental outcomes can be demonstrated by simulation of a virtual cell containing sub-diffraction sized (i.e. < 200 nm) lipid rafts (Fig. 6). Many researchers are interested in exploring the effects of phase separated regions within cells (e.g. lipid rafts at the plasma membrane and sub-cellular molecular condensates and granules). Here, we simulated small islands of lipid raft^44–48^ (10% of total surface area) with viscosity ten times higher than the surrounding membrane. Freely-diffusing, fluorescently-tagged, transmembrane proteins were then introduced into the model and they were allowed to move between the two lipid phases. Because molecules inside raft regions moved more slowly they became locally concentrated and our simulations revealed that raft locations could be identified in simulated TIRFM movies by generating a projected standard deviation of intensity image (SD image) (Fig. 6A&B compared to 6C)^11^. Model output also revealed a reduction in average mobility (from 0.102 to 0.056 µm^2^⋅s^−1^, Fig. 6D&E) because molecules showed a mixture of slow mobility within the rafts and faster mobility in the bulk membrane. However, MSD vs dT plots did not show anomalous diffusive behavior compared to MSD vs dT plots obtained from diffusion within pure lipid membrane because raft size was similar to the spatial resolution of video-rate tracking.

## Discussion

The model described uses computer memory to map a 3-dimensional volume containing a simulated biological cell bathed in extracellular medium. Each byte of allocated memory holds a number that is used to index a lookup table holding a rich set of physical–chemical properties which can have high complexity. Simulated molecules, treated as objects with specified attributes (in computing terms; classes, properties, and methods), can then roam within the volume either by diffusion (Brownian walk) or by ATP-driven, directed transport. We show how simple geometrical objects and more complex cellular structures can be created as voxelated volumes and how Brownian motion and motorized transport of individual molecules is simulated at each model time step using floating-point arithmetic. Because of the large spatial and temporal dynamic range various computational methods have been developed to allow “coarse-graining” to reduce limitations due to memory size (total model volume/(voxel unit size)^3^) and overall computation time, which is directly proportional to step-time (Δt) per iteration cycle. Logical tests performed at each iteration cycle to ensure molecules remained correctly localized to particular voxel volumes and interactions with other molecules obeyed simple kinetic and thermodynamic rules. It is important to note that computation time depends on the number of interacting molecules. The benchmark time for simulating 1,000 molecules of one class binding/unbinding to 1,000 molecules of another class was 57 s (1,000 time-steps, i7 PC), while for 10,000 by 10,000 molecules the simulation engine run-time increased to 56 min.

Model validation was performed first using membrane-bounded spheres, cubes and prolate ellipsoids which served as simple models to check the equations of motion and logical tests performed at each computational iteration cycle. Such tests revealed the important interaction between the experimental and simulated video imaging frame rate, computational step-time (Δt) and membrane curvature on reduced dimensionality due to projection of a 3-D volume onto a 2-dimensional imaging detector that integrates fluorescence signals over given time windows (i.e. camera exposure time).

We modeled thin membrane tubes within the cytoplasm (e.g. endoplasmic reticulum) and showed that, at the limit, molecular motion appeared unidimensional^11^, regardless of Δt because of the limiting voxel unit dimensions in the model and diffraction limitation (point spread function) of the simulated optical imaging system. The estimated diffusion coefficient, D, was underestimated unless corrections were made for reduced dimensionality.

Ability to model the complex cellular *milieu* was demonstrated by simulations of intra-filopodial transport, and cellular localization of the molecular motor myosin 10 which involved use of arbitrary shapes, random and directed molecular movements binding and unbinding to different structural elements. Finally, we demonstrated how large scale, dynamic structural rearrangements can be superimposed with the molecular simulations by investigating the fusion of intracellular vesicles to the plasma membrane and delivery of molecules from one membrane bilayer to another.

Further developments of this modeling approach are fairly straightforward to implement: For instance the plasma membrane can be made non-homogeneous by adding lipid rafts^48^ with lower or higher viscosity (as described above) likewise, sub-cellular, phase-separated granules and molecular condensates could also be created and defined within voxel volumes. The membrane could also be corralled and segmented according to the “picket-fence” model^18^ by adding rings of voxels in the membrane that can only be crossed in a probabilistic manner leading to so-called “hop-diffusion”.

We have shown how voxelated cellular structures can be constructed using mathematical algorithms^49^ or manual drawing, however, more realistic shapes defined by z-stacks of experimental images acquired via confocal^50^ or electron microscopy^51^ could also be created. One constraint is that cellular volumes and sub-structures should be fully enclosed with bounding perimeter surfaces that are thicker than one voxel to give compartment separation and ensure that molecules do not leak from one compartment to another erroneously. Fig. S1 in Supplementary Materials shows the results of a simulation based on topological data from Fig. 3C. The modelling approach is suited to simulating many aspects of cell biology and in particular could be valuable in simulating signaling pathways within and between cells. For instance immunological response networks, patches or sheets of cells representing organoids or cell-fate determination during development might be modelled and hypotheses tested. Because each voxel (byte) of computer memory in the simulated 3-D space acts as an index to a lookup table of physicochemical properties the complexity of the model can be readily expanded.

## Conclusion

The modeling approach reported here allows simulation of dynamic single molecule imaging experiments in live cells, which would be difficult to simulate using closed algebraic models, analytical or numerical methods. Use of Monte Carlo methods and a realistic 3-dimensional structural framework permits a wide variety of cell biological phenomena to be simulated and output as simulated (mock) video microscopy data sets that can be directly compared to real experimental data. We have tested and validated the modelling approach against experimental raw data sets obtained from our own laboratory and show how the modelling approach might be readily applied to other systems. New analysis and modelling approaches based on machine learning (ML) and generative AI networks (GANs) offer exciting possibilities in this field^52,53^. It is likely that traditional programming methods (described here) will assist in relating input and output to experimentally derived data sets and known ground truths and therefore help circumvent problems of model bias and hallucination which can occur with ML approaches.

## Supplementary Information

Supplementary Movie 1.

Supplementary Movie 2.

Supplementary Movie 3.

Supplementary Movie 4.

Supplementary Movie 5.

Supplementary Movie 6.

Supplementary Movie 7.

Supplementary Movie 8.

Supplementary Movie 9.

Supplementary Legends.

Supplementary Figure S1.

Supplementary Information 1.

## Data Availability

The executable version of this model, pre-defined scripts, and some results (image sequences) can be downloaded from www.mashanov.uk or www.github.com/GMashanov/GMvCell.

## Code availability

The full Borland C++ code is freely available for non-commercial use from www.github.com/GMashanov/GMvCell.
