# Supplementary material for: Single molecule dynamics in a virtual cell combining a 3-dimensional matrix model with random walks
Source: Sci Rep. 2024 Aug 28;14:20032. doi: 10.1038/s41598-024-70925-2 (PMC11358523; doi:10.1038/s41598-024-70925-2)
Supplement: Supplementary file 10 — Supplementary Legends. [file 41598_2024_70925_MOESM10_ESM.docx]

**Supplementary Material:**

**Figure S1 and model Settings screenshots. Figure S1 Artificial ER network.** A – The network was created in the horizontal plane at z=100 nm above the substrate using the record of GFP-KCNQ1 receptors shown on **Fig 3C to define the modeled virtual cell shape**. The tube diameter varies between 100 and 200 nm, thickness 3 voxels. B. The tube network was seeded with membrane proteins (D_lat_= 0.1 µm^2^ s^‑1^), and solution proteins (D= 2 µm^2^ s^‑1^). The results of the simulation are shown as an SD-projected image of the movie of membrane molecules on the left and intra-luminal molecules on the right. **Executable model (GMvCell.exe) Settings screenshots.**

**S2. File. Code examples.** Examples of rounded cell membrane, membrane thickening, creating set of vertices out of membrane voxels, tube fission and others.

**Movie S1. Construction of tubular network.** A Ø200 nm tube was seeded at a random position inside a small cell. It was allowed to grow in a randomly changing direction until it reached the cell membrane.

**Movie S2. Simulation of nucleus fission.** A cross-section of a small cell in a voxelated 3D space shows an elongated “nucleus” undergoing fission. Green balls represent membrane molecules diffusing in the nucleus membrane.

**Movie S3. Simulation of multimolecular interactions.** Rectangular cell 20×10×2 µm^3^ contained 500 membrane molecules, density 1 µm^-2^ (green) moving at a rate 0.2 µm^2^ s^-1^. These molecules can bind to each other forming clusters (on-rate 2x10^4^ s^-1^, off-rate 1 s^-1^). In the second half of the movie on-rate was reduced to 0 to initiate cluster dissociation. 1000 cytoplasm molecules, concentration 4.2 nM, K_diff_=2 µm^2^⋅s^-1^ (red) were allowed to bind membrane molecules (on-rate 1x10^6^ s^-1^, off-rate 0.2 s^-1^) during whole period of simulation.

**Movie S4.**  **Filaments growing in cytoplasm.** Rectangular cell 20×10×2 µm^3^ contained 20000 cytoplasm molecules, concentration 82 nM, fluorescent fraction 10%, K_diff_=2 µm^2^⋅s^-1^. Few molecules were allowed to form the seeds (get immobilized) which started the process of polymerization – cytoplasm molecules can bind to the growing end of each filament, pitch size 8 nm, on-rate 1x10^6^ s^-1^, off-rate 0.01 s^-1^). The grow rate slowed down to zero at the second part of the movie because >99% of the molecules were polymerized.

**Movie S5. Image sequences of fluorescent membrane proteins moving in the curved plasma cell membranes.** See main text and **Fig 2**. The heat maps are shown at the end of the video.

**Movie S6. Image sequence of fluorescent membrane molecules (green) and intra-lumen molecules (red) moving in the ER membranes and tube lumen.** The right half of the ER network was photobleached at t= 0.5 s to demonstrate the fluorescence recovery after photobleaching at the single molecule level. Scale bar 5 µm. See the main text for the details of this simulation.

**Movie S7. Simulation of myosin-10 accumulation at the tips of filopodia.** Molecules of myosin-10 (green spots) randomly diffuse in the cell body before switching to directed movement in filopodia. The red color is used to visualize the cell membrane. See the main text for the details of this simulation.

**Movie S8** **Vesicle movements in a small cell**. (left) shows the sequence of images simulating vesicle movements in a virtual *Schizosaccharomyces Pombe* yeast cell. The cell also contains “red” S-B molecules in the cytoplasm used to visualize cell volume. Video data recorded from a real *S. Pombe* cell is shown on the right for comparison - the cell was transfected with calmodulin-2-GFP, associated with vesicle membrane and calmodulin-1-mCherry diffusing in the cytoplasm (See Baker *et al.*, (30)

**Movie S9** **Simulation of exocytosis.** Fusion dynamics tested using an Ø50 nm tube (left) and instant transfer (right) fusion conditions.
