# Supplementary material for: Single molecule dynamics in a virtual cell combining a 3-dimensional matrix model with random walks
Source: Sci Rep. 2024 Aug 28;14:20032. doi: 10.1038/s41598-024-70925-2 (PMC11358523; doi:10.1038/s41598-024-70925-2)

**Fig. S1.** **Artificial ER network:** **Upper panel)** The network was created in a horizontal plane located at z = 100 nm above the microscope coverslip using the record of GFP-KCNQ1 receptors shown on **Fig. 3C**. The tube diameter varies between 100 and 200 nm, thickness 3 voxels. **Lower Panel)** The tube network was seeded with membrane-bound and luminal space single molecules (D = 0.1 µm^2^ s^‑1^ and 2 µm^2^ s^‑1^, respectively). Results are shown as SD-projected image of membrane bound molecules (**Left**) and molecules moving within the luminal space (**Right**).

Executable model “Settings” screenshots (See GMvCell Help file for detailed explanation of each menu element).


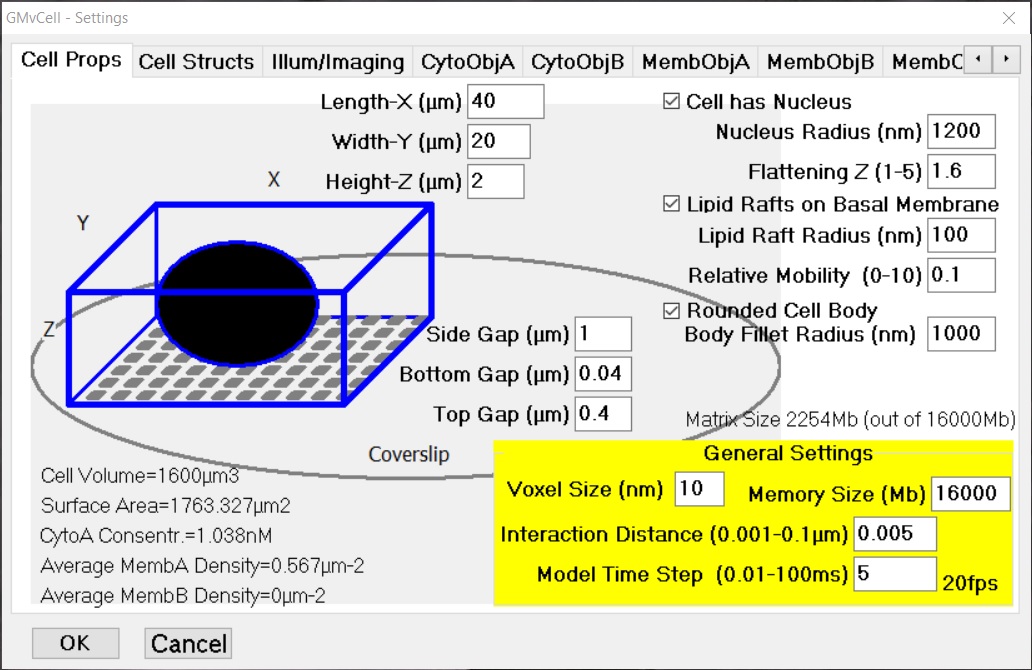


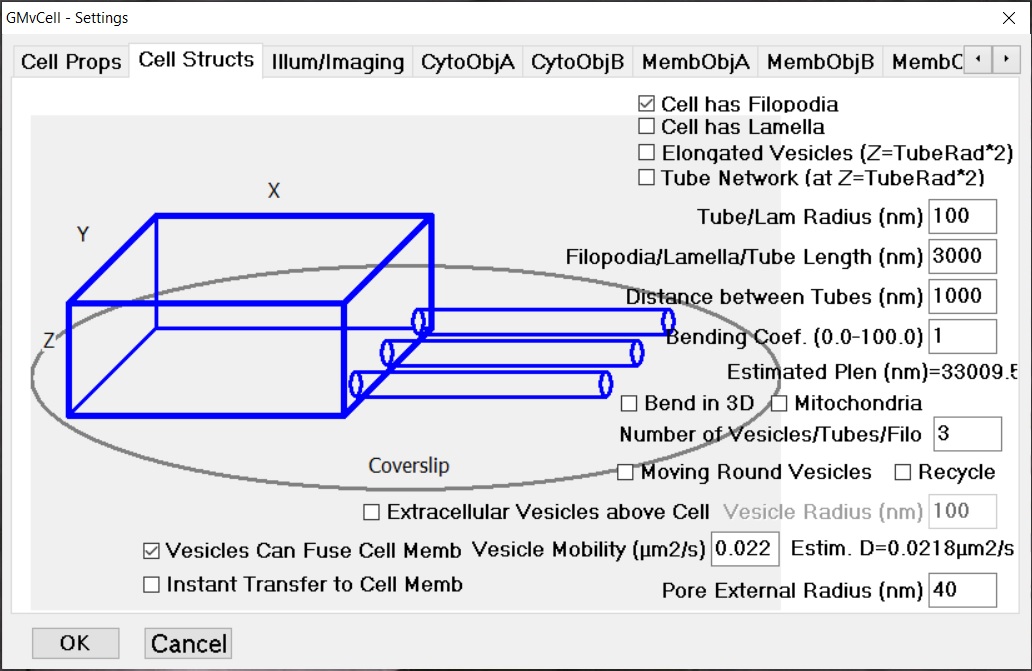


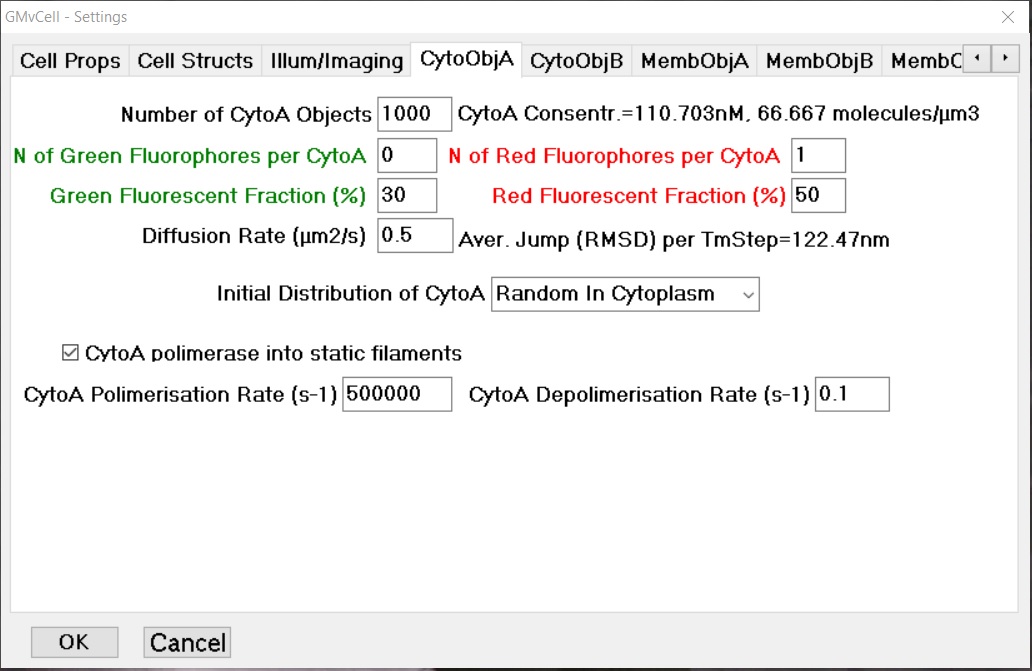


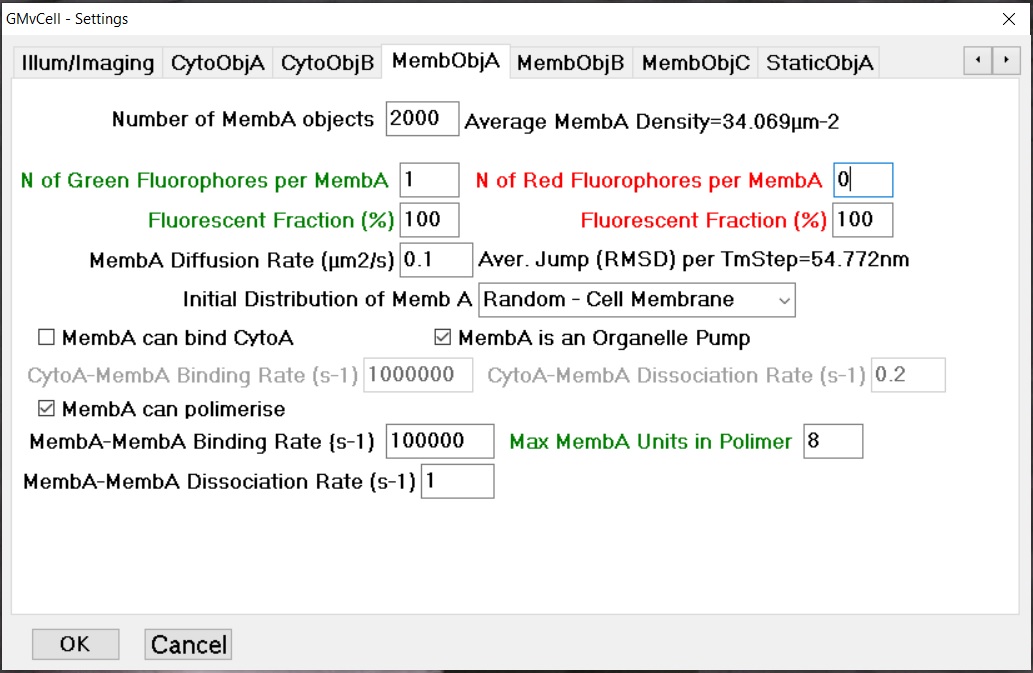


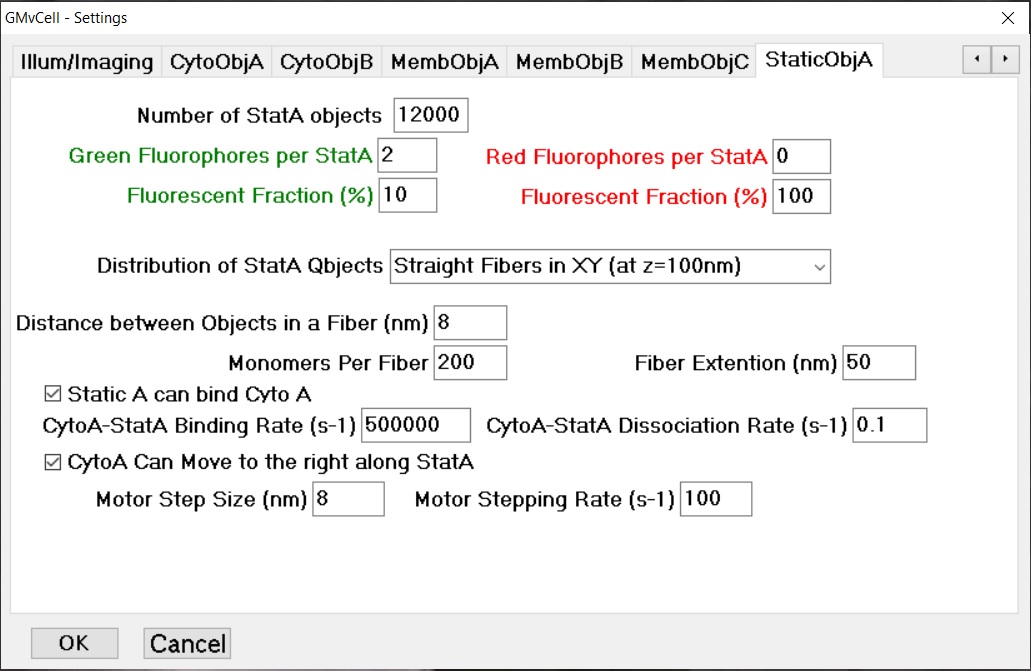


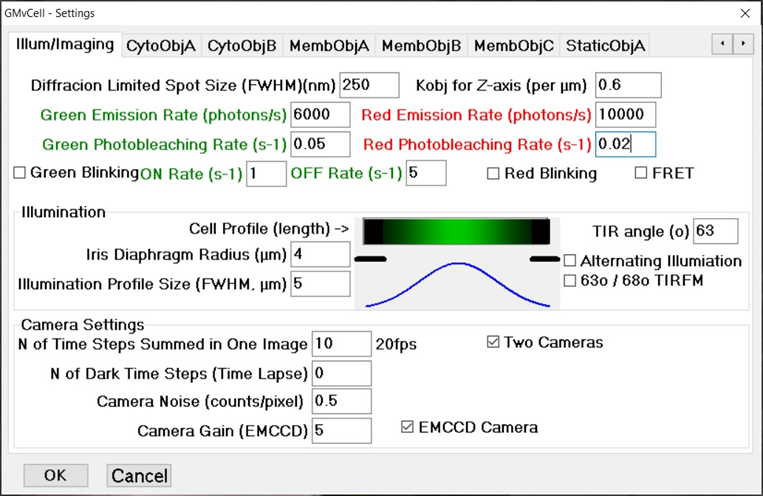

Supplement: Supplementary file 11 — Supplementary Figure S1. [file 41598_2024_70925_MOESM11_ESM.docx]
