# Supplementary material for: Single molecule dynamics in a virtual cell combining a 3-dimensional matrix model with random walks
Source: Sci Rep. 2024 Aug 28;14:20032. doi: 10.1038/s41598-024-70925-2 (PMC11358523; doi:10.1038/s41598-024-70925-2)
Supplement: Supplementary file 12 — Supplementary Information 1. [file 41598_2024_70925_MOESM12_ESM.pdf]

Some practical tips:

1. The model has a modular structure – some parts (e.g. fluorescent output or interactions between molecules) can be omitted for a particular test.
2. A bigger voxel size will substantially increase the speed of initialization and model run if you do large volume/cell simulation.
3. Initial random distribution of molecules belonging to small compartments (e.g. vesicles) in a large volume can be achieved by placing these molecules at known position in each structure and allowing it to diffuse for some time to achieve a steady-state random distribution.
4. Flat parts of membrane can be 1 voxel thick (Code Example 1).
5. Each position of a moving molecule is calculated in 3D space regardless of the type of molecule, but, unlike solution-type molecules (Example Code 5) the "voxel" path of membrane-type molecules are checked separately in each dimension (Example Code 6) to ensure the molecule remains in the membrane space. This approach allows us to use long time-steps in case of large flat membrane structures (Code Example 1).
6. It is important to adjust the model time-step (Δt) so that the average displacement, RMSD=$\surd$(2D*ΔT) for membrane molecules (which have much slower diffusion that cytosolic molecules) is close to the voxel size so that molecules remain within tightly curved membrane structures.
7. Trans-membrane transport of solution molecules can be modelled by releasing solution molecules, bound to membrane molecules, to a different medium voxel (e.g. cytoplasm-extracellular or cytoplasm-nucleoplasm). See Code Example 9.
8. The same function (Code Example 9) is used to move membrane molecules during formation of a pore between vesicle and plasma membrane (Fig. 5B) and during organelle fission (Movie S7 and Code Example 10).

Note:

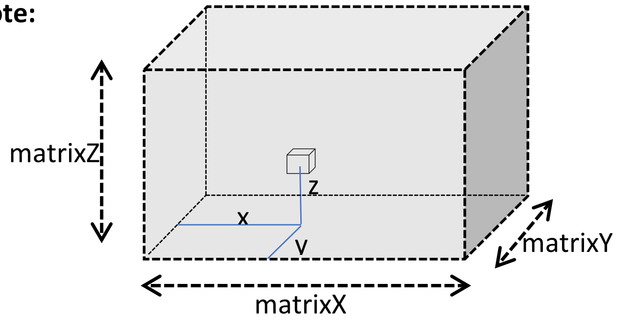

Explain array indexing is language specific:
For 3-D array indexing : matrix[x][y][z]
For 1-D array indexing : matrixXY=matrixX*matrix
matrixSize = matrixXY*matrixZ
x,y,z, index = matrix[x+(y*matrixX)+(z*matrixXY)]

Define "byte" variables used to index physico-chemical table
e.g. (I have given random numbers!)
CYTOPLASM = 10
LUMEN = 33
CELL_MEMB = 200
CYTOPLASM = 150
VESI_MEMB = 127

Code Example 1:

```
//- Building a simple rectangular shell (See Code Example 7 for beveled shell)
for ( __int64 z=bot; z < top; z++ )
  for ( __int64 y=up; y < down; y++ )
    for ( __int64 x=left; x < right; x++ )
          matrix[x+(y*matrixX)+(z*matrixXY)]= __byte CELL_MEMB;
```

```
for ( __int64 z=bot+1; z < top-1; z++ )
  for ( __int64 y=up+1; y < down-1; y++ )
    for ( __int64 x=left+1; x < right-1; x++ )
        matrix[x+(y*matrixX)+(z*matrixXY)]= __byte CYTOPLASM;

// Note: Membrane thickness 1 voxel, matrixX and matrixXY are x and x*y
// dimensions of the matrix.
// 64-bit variables must be used to address arrays > 2Gvoxels.
```

Code Example 2:

```
//------------    Thickening membranes ---------------------------------
void Thickening(void)
{ byte voxVal;
  for ( __int64 z=1; z < matrixZ-1; z++ )
     { for ( __int64 y=1; y < matrixY-1; y++ )
           for ( __int64 x=1; x < matrixX-1; x++ )
              if ( (voxVal=matrix[x+(y*matrixX)+(z*matrixXY)]) > VESI_MEMB )
                 { for ( __int64 zz=z-1; zz < z+2; zz++ )
                      for ( __int64 yy=y-1; yy < y+2; yy++ )
                         for ( __int64 xx=x-1; xx < x+2; xx++ )
                            if ( matrix[xx+(yy*matrixX)+(zz*matrixXY)] < 200 )
                              matrix[xx+(yy*matrixX)+(zz*matrixXY)]=voxVal-100;
           }
     }
  for ( __int64 i=0; i < matrixSize; i++ )  // restore original voxel values
    if ( matrix[i] < 200 && matrix[i] > 100 ) matrix[i]+=100;
}
```

Code Example 3:

```
//---- Flatten vesicle in a free space at cX, cY, cZ center  ------
void MakeVesicle(int cX,int cY,int cZ,int rad,float shape,int thickness,byte membType,byte lumenType)
{ float dX,dY,dZ,r;
  for ( __int64 z=cZ-rad; z < cZ+rad; z++ )
     for ( __int64 y=cY-rad; y < cY+rad; y++ )
        for ( __int64 x=cX-rad/shape; x < cX+rad/shape; x++ )
           { dX=(x-cX)*shape; dY=y-cY; dZ=z-cZ;
             r=sqrt(dX*dX+dY*dY+dZ*dZ); // square root of 3D vector
             if ( r <= rad )
                matrix[x+(y*matrixX)+(z*matrixXY)]=membType;
             if ( r < rad-thickness )
                matrix[x+(y*matrixX)+(z*matrixXY)]=lumenType;
           }
}
// Note: __int64 variables should be used to address large
// array in computer memory. The ellipticity is set by
// variable "shape", membrane thickness by "thickness".
// See nucleus example on Fig 1D.
```

Code Example 4:

```
// This function is used to build tubes and other structures (Fig. 3A, Movie S1)
void FillSphere(int rad,int cX,int cY,int cZ,int shell,int lumen,int noGo)
{ __int64 absX,absY,absZ;  int val=0;
  for ( int x=-rad-1,r; x <= rad+1; x++ )  //
```

```
    for ( int y=-rad-1; y <= rad+1; y++ )
      for ( int z=-rad-1; z <= rad+1; z++ )
        { absX=cX+x;absY=cY+y;absZ=cZ+z;
          if ( absX < 0 || absY < 0 || absZ < 0 || absX >= matrixX ||
               absY >= matrixY || absZ >= matrixZ ) continue; // out of scope
           r=sqrt((x*x)+(y*y)+(z*z));
           val=matrix[absX+(absY*matrixX)+(absZ*matrixXY)];
           if ( r <= rad && val != noGo && val != lumen )
                matrix[absX+(absY*matrixX)+(absZ*matrixXY)]=shell;
           if ( r < rad-1 && val != noGo )
              matrix[absX+(absY*matrixX)+(absZ*matrixXY)]=lumen;
         }
}
// Note: All variables have voxel units, cX, cY, cZ – center
// of the sphere of radius rad, r is distance from the center
// aX, aY, aZ are absolute coordinates of current voxel
```

Code Example 5:

```
// this function is used to check if the path of solution molecule is free from
// obstacles (membrane voxels) – it stops at last valid voxel
void CheckPathC(float x,float y,float z,float *dX,float *dY,float *dZ)
{ int steps=abs(*dX/nmVox)+2;
  if ( abs(*dY/nmVox)+2 > steps ) steps=abs(*dY/nmVox)+2;
  if ( abs(*dZ/nmVox)+2 > steps ) steps=abs(*dZ/nmVox)+2;
  float stepXnm=*dX/steps,stepYnm=*dY/steps,stepZnm=*dZ/steps;
  __int64 xVox,yVox,zVox;  // 64 bit integer variables
  *dX=*dY=*dZ=0.0;
  for ( int i=1; i <= steps; i++ )
    { x+=stepXnm; y+=stepYnm; z+=stepZnm;
     xVox=x/nmVox; yVox=y/nmVox; zVox=z/nmVox;
     if ( matrix[xVox+(yVox*matrixX)+(zVox*matrixXY)] < 10 )
       { *dX=stepXnm*i; *dY=stepYnm*i; *dZ=stepZnm*i; }
     else return; // terminate at last solution voxel.
    }
}
// Note: SM coordinates in floating-point (nanometre) units converted into
// matrix/voxel coordinates (__int64) units using nmVox scaler (e.g.,
// 5nm/vox). *dX,*dY,*dZ are pointers used to pass new position into
// function and to return "path-checked" new position of the object.
```

Code Example 6:

```
//---Check molecule path in membrane (separately in each axis) -------------
void CheckPathM(float x,float y,float z,float *dX,float *dY,float *dZ)
{ __int64 xVox=x/nmVox,yVox=y/nmVox,zVox=z/nmVox;
  int stepsX=abs(*dX/nmVox)+2,stepsY=abs(*dY/nmVox)+2,stepsZ=abs(*dZ/nmVox)+2;
  float stepXnm=*dX/stepsX,stepYnm=*dY/stepsY,stepZnm=*dZ/stepsZ;
  *dX=*dY=*dZ=0.0;
  for ( int i=1; i <= stepsX; i++ )
     { x+=stepXnm; xVox=x/nmVox;
       if ( matrix[xVox+(yVox*matrixX)+(zVox*matrixXY)] > 99 ) *dX=stepXnm*i;
       else { x-=stepXnm; break; } // 1 step back
    }
```

```
  xVox=x/nmVox;
  for ( int i=1; i <= stepsY; i++ )
    { y+=stepYnm; yVox=y/nmVox;
      if ( matrix[xVox+(yVox*matrixX)+(zVox*matrixXY)] > 99 ) *dY=stepYnm*i;
      else { y-=stepYnm; break; } // 1 step back
    }
  yVox=y/nmVox;
  for ( int i=1; i <= stepsZ; i++ )
    { z+=stepZnm; zVox=z/nmVox;
     if ( matrix[xVox+(yVox*matrixX)+(zVox*matrixXY)] > 99 ) *dZ=stepZnm*i;
     else break;
    }
}
```

Code Example 7:

```
//---   Rounded shell (ALL UNITS ARE VOXELS)  -------------------
void MakeRoundedShell(int X1,int X2,int Y1,int Y2,int Z1,int Z2,int rad)
{ int cellTop=Z2,cellRight=Y2,cellDown=Y2;
  int x1=X1+rad,x2=X2-rad,y1=Y1+rad,y2=Y2-rad,z1=Z1+rad,z2=Z2-rad;
  for ( __int64 z=Z1,dX,dY,dZ,dist; z <= Z2; z++ ) // make borders
    for ( __int64 y=Y1; y <= Y2; y++ )
      for ( __int64 x=X1; x <= X2; x++ )
        if ( z < z1 || y < y1 || x < x1 || z > z2 ||  y > y2 || x > x2 )  // rounded parts of the shell
          { dX=0; dY=0; dZ=0;
            if ( z < z1 ) dZ=(z1-z)*0.99;
            if ( z > z2 ) dZ=(z-z2)*0.99;
            if ( y < y1 ) dY=(y1-y);
            if ( y > y2 ) dY=(y-y2);
            if ( x < x1 ) dX=(x1-x);
            if ( x > x2 ) dX=(x-x2);
            dist=sqrt(dX*dX+dY*dY+dZ*dZ);
            if ( dist < rad ) matrix[x+(y*matrixX)+(z*matrixXY)]=CELL_MEMB;
            if ( dist < rad-1 && z != botGapVox && z != botGapVox+1 && z != cellTop )
              matrix[x+(y*matrixX)+(z*matrixXY)]=CYTOPLASM;
          }
        else matrix[x+(y*matrixX)+(z*matrixXY)]=CELL_MEMB;
  for ( __int64 z=z1; z < z2+1; z++ ) // central cavity
   for ( __int64 y=y1; y < y2+1; y++ )
    for ( __int64 x=x1; x < x2+1; x++ )
      matrix[x+(y*matrixX)+(z*matrixXY)]=CYTOPLASM;
}
```

Code Example 8:

```
// Create array of vertices mapping membrane voxels linked to neighboring voxels
bool SetVertices(void)
{ int index=0,nLim,curZ=0,first,last,layer2first,layer2last,layersNum=0;
  for ( int i=0; i < 10000; i++ ) vertZind[i]=0;
  for ( __int64 z=0; z < matrixZ && index < VERT_LIM; z++ )
   { for ( __int64 y=0; y < matrixY && index < VERT_LIM; y++ )
      for ( __int64 x=0; x < matrixX && index < VERT_LIM; x++ )
           if ( matrix[x+(y*matrixX)+(z*matrixXY)] > 100 )
            { vert[index].x=x; vert[index].y=y;vert[index].z=z; // coordinates
```

```
            for ( int j=0; j < 15; j++ ) vert[index].nInd[j]=-1; // no index
                if ( z > curZ ) { curZ=z; vertZind[z]=index; layersNum++; } // at z
                vert[index].type=matrix[x+(y*matrixX)+(z*matrixXY)];
                index++;
            }
  }
 vertNum=index; vertZind[curZ+1]=vertNum; // last layer
 if ( vertNum == 0 )
  { ShowMessage("CRITICAL ERROR - No vertices"); return false; }
 if ( vertNum >= VERT_LIM )
  { ShowMessage("ERROR – TOO MANY VERTICES"); return false; }
 // connecting vertices
 for (int i=0; i < 3; i++ ) layer[i] = new int[matrixXY];  // create 3 layers
 if ( layer[0] == NULL || layer[1] == NULL || layer[2] == NULL )
       { ShowMessage("Layer allocation ERROR"); return false; }
 for ( int i=0; i < matrixXY; i++ ) layer[0][i]=layer[1][i]=layer[2][i]=-1; // zeroing
 // before Search layer 2 filled with initial Z
 curZ=vert[0].z;
 int maxCon=0;
 layer2first=vertZind[curZ];layer2last=vertZind[curZ+1];
 for ( int i=layer2first,xx,yy; i < layer2last; i++ )
       { xx=vert[i].x; yy=vert[i].y; layer[2][xx+(yy*matrixX)]=i; }
 // main connecting cycle
 for ( int i=0; i < layersNum; i++,curZ++ )
  { for ( int i=0; i < matrixXY; i++ ) // shift layers
        { layer[0][i]=layer[1][i]; layer[1][i]=layer[2][i]; layer[2][i]=-1; }
   layer2first=vertZind[curZ+1],layer2last=vertZind[curZ+2];
   maxCon=0;
   for ( int i=layer2first,xx,yy; i < layer2last; i++ ) // fill layer 2
    { xx=vert[i].x; yy=vert[i].y; layer[2][xx+yy*matrixX]=i; }
     // find first and last vertex in a layer
     first=vertZind[curZ];last=vertZind[curZ+1]; //indexing
     for ( int j=first,x,y; j < last; j++ )
       { x=vert[j].x; y=vert[j].y; nLim=-1;
        for ( int zz=0,ind; zz < 3; zz++ ) // search for membrane voxels
          for ( int yy=y-1; yy < y+2; yy++ )
            for ( int xx=x-1; xx < x+2; xx++ )
              { ind=layer[zz][xx+(yy*matrixX)];
               if ( ind > -1 && ind != j && vert[j].type == vert[ind].type && nLim < 14 )
                { nLim++; vert[j].nInd[nLim]=ind;
                  if ( nLim > maxCon ) maxCon=nLim;
                }
              }
            }
     }
 return true; // READY for running!
}
```

Code Example 9:

```
//-Checks if object is in a correct voxel (medium). If not, moves it to
// a nearest voxel of correct type which is found using random number generator
bool MoveToNearestVoxel(Obj *obj,int rad,int medium)
{ int cX=obj->x/nmVox,cY=obj->y/nmVox,cZ=obj->z/nmVox,distance=100000000;
```

```
  int span=rad*2,min=0;
  __int64 x=cX,y=cY,z=cZ; float hVox=(float)nmVox*0.5; bool result=false;
  if ( matrix[x+(y*matrixX)+(z*matrixXY)] == medium ) return true; // no move
  for ( int i=0,good=0; i < 1000 && good < 10; i++ ) // up to 1000 attempts
   { x=cX+random(span)-rad;y=cY+random(span)-rad;z=cZ+random(span)-rad;
     if ( x < 0 || y < 0 || z < 0 ) continue; // check scope
     if ( x >= matrixX || y >= matrixY || z >= matrixZ ) continue;
     if ( matrix[x+(y*matrixX)+(z*matrixXY)] == medium )
     if ( (min=sqrt((x-cX)*(x-cX)+(y-cY)*(y-cY)+(z-cZ)*(z-cZ))) < distance )
        { obj->x=(x*nmVox)+hVox; obj->y=(y*nmVox)+hVox;obj->z=(z*nmVox)+hVox;
          distance=min; result=true; good++; // limit to 10 good voxels
        }
   }
  return result;
}
```

Code Example 10:

```
// For nucleus fission elongate round vesicle by at least 2rad (tubeLenVox) before running
// The separation is controlled by "stage" value (0-1.0).
// use code example 9 to update objects locations inside, outside and in membrane, See Movie S2
void TubeFission(int rad,int cXvox,int cYvox,int cZvox,float stage)
{ int dX,dY,dZ,rrr;
  int beg=cXvox-(tubeLenVox*0.5),end=cXvox+(tubeLenVox*0.5);
  for ( __int64 x=beg; x < end; x++ )
    for ( __int64 y=cYvox-rad-2; y < cYvox+rad+2; y++ )
      for ( __int64 z=cZvox-rad-2; z < cZvox+rad+2; z++ )
          { dY=cYvox-y; dZ=cZvox-z;
            rrr=sqrt(dY*dY+dZ*dZ);  // round end
            if ( x < beg+rad ) dX=((beg+rad)-x); // left round part
            if ( x > end-rad ) dX=((end-rad)-x); // right round part
            if ( x > beg+rad && x < cXvox )  dX=((beg+rad)-x)*stage;
            if ( x < end-rad && x >= cXvox ) dX=((end-rad)-x)*stage;
            rrr=sqrt(dY*dY+dZ*dZ+dX*dX);
            matrix[x+(y*matrixX)+(z*matrixXY)]=CYTOPLASM; // replace all voxels
            if ( rrr < rad )   matrix[x+(y*matrixX)+(z*matrixXY)]=VESI_MEMB;
            if ( rrr < rad-3 ) matrix[x+(y*matrixX)+(z*matrixXY)]=LUMEN;
          }
}
//---------------------------------------------------------------------------
```
